# Concurrent use of medicines with similar adverse drug reaction profiles in older adults: identifying potential deprescribing opportunities using population-level dispensing data

**DOI:** 10.1007/s11096-026-02148-6

**Published:** 2026-04-21

**Authors:** Imaina Widagdo, Tien Bui, Lauren Cortis, Andre Andrade, Elizabeth Roughead

**Affiliations:** Quality Use of Medicines and Pharmacy Research Centre, School of Pharmacy and Biomedical Science, College of Health, Adelaide University, Adelaide, Australia

**Keywords:** Aged, Deprescriptions, Drug-related side effects and adverse reactions, Drug utilization, Pharmacoepidemiology, Polypharmacy

## Abstract

**Introduction:**

Older adults are more vulnerable to medicine-related harm. Identifying concurrent use of medicines with similar adverse drug reaction (ADR) profiles may support opportunities for safer prescribing and deprescribing.

**Aim:**

To examine the frequency of concurrent use of medicines with overlapping ADR profiles in older adults. A secondary aim was to examine the contribution of potentially inappropriate medicine (PIMs) to overlapping ADR profiles.

**Method:**

We used data from the 10% sample of dispensing claims from the Australian Pharmaceutical Benefits Scheme between April and June 2022. Medicines were classified by ADR risk using an adapted version of the 2018 Scottish cumulative toxicity tool, which identifies 13 ADR types based on product information. PIMs were identified using the 2024 Australian PIMs list. Individuals aged 65 years or older who were dispensed at least one medicine associated with a listed ADR were included. The outcome of interest was the percentage of persons with an overlapping ADR (dispensed at least two medicines associated with the same ADR). A secondary analysis examined changes in overlapping ADR profiles under a hypothetical scenario excluding PIMs.

**Results:**

Our analysis included 323,599 older adults, of which 75.5% (n = 244,331) had at least one overlapping ADR risk, while 64% (n = 206,815) had two or more overlapping ADR risks. Falls/fractures 70.1% (n = 226,873), constipation 53.5% (n = 173,287), and renal injury 38.5% (n = 124,471) were the most common overlapping ADR risks. Under a hypothetical scenario excluding PIMs, an additional 13,803 people would have no falls/fracture overlapping ADR risk, and 19,396 would have no overlapping ADR risk.

**Conclusion:**

Concurrent use of medicines with similar ADR profiles is common among older adults. Structured tools to identify the use of medicines with similar ADR risks may support deprescribing and safer medicine use in this population.

## Impact statements

- Identifying overlapping ADR profiles from concurrent medicines may support safer prescribing and deprescribing in older adults.
- Embedding structured ADR-risk profiling tools into medicines review may help pharmacists and clinicians prioritise high-risk combinations, reduce preventable harm, and improve patient outcomes.
- Data-driven screening approaches may help strengthen clinical pharmacy practice by identifying potential deprescribing opportunities and supporting more sustainable use of healthcare resources.

## Introduction

Medicines provide many benefits in treating and preventing health problems, but they also carry risks, particularly for older adults (aged 65 years and older). This population is often more sensitive to medicines and are disproportionately affected by medicine-related harms [1]. This can be due to altered physiology (relative to younger adults) which changes the way medicines are metabolised and excreted from the body, most importantly reduced renal and hepatic function, which increases the risk of adverse effects. Additionally, frailty and comorbidities can further increase susceptibility to medicine-related harm [2]. Harm from medicines is frequent; a 2022 Australian systematic review found that 250,000 hospital admissions each year in Australia were related to medicine related problems and that two-thirds of these are potentially preventable [3].

Certain medicine classes, including anticholinergics, antipsychotics, diuretics, antidepressants, opioids and non-steroidal anti-inflammatory medicines have a high risk of adverse drug reactions, and this risk may be compounded when such medicines are used together [4, 5]. Assessing and managing the risks associated with concurrent medicines use is an important role for clinicians to optimise patient outcomes. Deprescribing, defined as the systematic, patient-centred process of tapering, reducing, or stopping medicines, is especially important for medicines that no longer provide therapeutic benefit or that potentially increase the risk of adverse events above benefit [6, 7].

In 2022, one in two (51%) Australians aged 65–74 years and 72% of those aged 75 years and older were taking five or more medicines concurrently [8]. Internationally, polypharmacy is also increasingly common, although prevalence estimates vary widely. A 2024 systematic review of 49 studies across clinical and community settings reported that between 2.6 and 86.6% of older adults worldwide were using five or more medicines [9]. Given the substantial exposure to multiple medicines, potentially inappropriate medication (PIM) use among older adults is also common. A 2023 systematic review and meta-analysis including 371.2 million older adults from 17 countries reported a pooled prevalence of PIM use of 36.7%, with evidence that PIM use among older adults has increased over the past two decades [10]. As concurrent medicine use becomes more prevalent among older adults [8–12], understanding the risk of adverse drug reactions (ADRs) from concurrent medicine use and the potential benefits of deprescribing may be helpful. Prior research has largely focused on estimating the occurrence of observed ADRs rather than identifying the extent of concurrent use of medicines with overlapping ADR profiles, highlighting an important knowledge gap. Addressing this gap may help inform safer prescribing and deprescribing strategies in clinical practice.

### Aim

This study aimed to examine the frequency of concurrent use of medicines with similar ADR profiles in older adults. A secondary aim was to examine the contribution of medicines identified on explicit PIM lists for older people to overlapping ADR profiles, as a means of identifying potential deprescribing opportunities.

## Method

### Data source and study population

This study used the Australian Pharmaceutical Benefits Scheme (PBS) 10% dataset. The PBS 10% dataset contains records of all PBS-subsidised medicines dispensed to a 10% random sample of Medicare-eligible Australian residents. It does not include over-the-counter medicines, or medicines administered in hospitals. The analysis included dispensing records of older Australians who were supplied one or more medicines between April and June 2022. The April to June observation window was selected to minimise potential influence of the PBS Safety Net (a scheme that lowers or removes medicine costs after a spending limit is reached) and associated stockpiling later in the calendar year.

### Medicines use definition

An adapted version of the 2018 Scottish cumulative toxicity tool, which includes 13 ADR risks, was used to identify medicines with similar ADR profiles [13]. This tool cross-tabulates medicines and associated ADR types to identify potential cumulative effects where multiple medicines contribute to the same ADR. ADR profiles were based on commonly recognised and preventable adverse effects, informed by Summary of Product Characteristics/Product Information (SPC/PI) and pharmacological mechanisms [13]. The ADR types included, for example, falls and fractures, bleeding, constipation, and urinary retention.

For each ADR profile, we calculated the number of people dispensed two or more medicines with the same ADR listed in the product information for each medicine (termed overlapping ADR risk), where medicines were considered concurrent if dispensed at least once within the April to June 2022 observation window.

Medicines were coded in the dataset according to the World Health Organization (WHO) Anatomical and Therapeutic Chemical (ATC) Classification [14]. Potentially inappropriate medicines (PIMs) were identified based on the 2024 Australian potentially inappropriate medicine list [15]. The number of medicines used was calculated based on the fifth -level ATC code, with combination medicines counted based on their individual components. Only unique medicines dispensed within the analysis period (April-June 2022) were counted for each individual.

### Statistical analysis

The outcome of interest was the percentage of persons with an overlapping ADR (dispensed at least two medicines associated with the same ADR). Descriptive statistics were used to summarise participant characteristics, including age group, sex, number of concurrent medicines with potential ADR risk, and PIM use. Frequencies and proportions were reported with 95% confidence intervals, calculated using the Wald method [16]. Analyses were conducted using SAS software, version 9.4 (SAS Institute Inc., Cary, NC, USA).

A secondary descriptive analysis was undertaken to examine the potential contribution of PIM to overlapping ADR profile. For this analysis, the number of medicines contributing to specific ADR profiles and the total number of overlapping ADR types per person were calculated under two scenarios: including all medicines and excluding medicines classified as PIMs. This analysis was intended to illustrate differences in ADR-related medicine exposure under a hypothetical deprescribing scenario, rather than to estimate causal effects. Absolute change (percentage points) was calculated as the difference in the prevalence of concurrent medicines with overlapping ADR profiles between scenarios including and excluding PIMs. Relative change was calculated as the difference between scenarios divided by the value in the reference scenario (including PIMs), expressed as a percentage.

The analysis also included mapping the redistribution of individuals across categories defined by (i) the number of medicines associated with falls or fracture ADRs and (ii) the total number of overlapping ADR types per person, comparing scenarios including and excluding PIMs.

### Ethics approval

The study and publication were approved by the Services Australia External Request Evaluation Committee (EREC) (Approval Number RMS 3483, 4 February 2024).

## Results

A total of 323,599 older people were dispensed at least one medicine with the specified ADR listed in product information (Table 1). Just over half (n = 165,627) of the population were aged 65–74 years (51.2%, 95% CI 51–51.4), and 53.5% (n = 172,983) were females (95% CI 53.3–53.6). Overall, 244,331 individuals (75.5%, 95% CI 75.4–75.7) had at least one overlapping ADR risk, while 206,815 (64%) had two or more overlapping ADR risks. The most common ADR risks with concurrent medicine use (at least two medicines concurrently) identified were falls and fractures (70.1%, n = 226,873, 95% CI 69.95–70.27), constipation (53.5%, n = 173,287, 95% CI 53.4–53.7), and renal injury (38.5%, n = 124,471, 95% CI 38.3–38.6).

**Table 1:** Characteristics of study population (N = 323,599)

Characteristics	Number of people	%	95% Confidence interval
Age group			
65–74	165,627	51.2	(51.01–51.36)
75–84	116,873	36.1	(35.95–36.28)
85+	41,099	12.7	(12.59–12.82)
Sex			
Female	172,983	53.5	(53.28–53.63)
Male	150,616	46.5	(46.37–46.72)
Total number of medicines with potential Adverse Drug Reaction (ADR)			
1	79,268	24.5	(24.35–24.64)
2–3	142,904	44.2	(43.99–44.33)
4–5	69,975	21.6	(21.48–21.77)
≥ 6	31,452	9.7	(9.62–9.82)
Total ADR types from concurrent use of medicines			
0	89,615	27.7	(27.54–27.85)
1	27,169	8.4	(8.3–8.49)
2	71,051	22	(21.81–22.1)
3	66,343	20.5	(20.36–20.64)
4	29,808	9.2	(9.11–9.31)
5	18,045	5.6	(5.5–5.66)
≥ 6	21,568	6.7	(6.58–6.75)
Most common overlapping ADR profiles (top 5)			
Falls/fracture	226,873	70.1	(69.95–70.27)
Constipation	173,287	53.5	(53.38–53.72)
Renal injury	124,471	38.5	(38.3–38.63)
Central nervous system depression	61,343	19	(18.82–19.09)
Bleeding	29,490	9.1	(9.01–9.21)
Potentially inappropriate medicine (PIM)			
At least one PIM	109,942	34	(33.81–34.14)

Reductions in the number of people with concurrent medicines were observed across all ADR types following the hypothetical removal of PIMs (Table 2). The largest absolute reductions were seen for central nervous system depression (n = 25,593), constipation (n = 22,741), and urinary retention (n = 22,186). In relative terms, the largest percentage reductions were observed for hypoglycaemia, serotonin syndrome, and urinary retention, although these occurred in ADR types with smaller numbers of people in the scenario including PIMs (Table 2).

**Table 2:** Reduction in number of people with ≥ 2 concurrent medicines with overlapping ADR profiles under a hypothetical potentially inappropriate medicine (PIM) deprescribing scenario

ADR types	Number of people with ≥ 2 concurrent medicines (including PIM)	Reduction in number of people with ≥ 2 concurrent medicines (excluding PIM)		
		Number of people	Absolute change (percentage points)	Relative change (%)
Falls/fractures	226,873	13,803	4.3%	6.1%
Constipation	173,287	22,741	7.0%	13.1%
Renal injury	124,471	13,258	4.1%	10.7%
Central nervous system depression	61,343	25,593	7.9%	41.7%
Bleeding	29,490	12,821	4.0%	43.5%
Urinary retention	27,376	22,186	6.9%	81.0%
Heart failure	27,277	15,203	4.7%	55.7%
Serotonin syndrome	18,245	16,355	5.1%	89.6%
Hypokalemia	9,470	1,417	0.4%	15.0%
Bradycardia	8,514	1,533	0.5%	18.0%
Hypoglycaemia	7,536	7,172	2.2%	95.2%
Hyperkalemia	7,263	0	0.0%	0.0%
Glaucoma	3,528	2,808	0.9%	79.6%

*PIM* potentially inappropriate medicine

If PIMs were ceased, the number of older people with concurrent medicines associated with ADR risk of falls or fracture would decrease by 13,803 (Table 2). This corresponds to a reduction from 70.1% to 65.8% (4.3 percentage points, 6.1% relative, n = 13,803) (Table 2).

The distribution of changes in the number of concurrent medicines associated with falls or fracture ADR risk under hypothetical PIM removal is shown in Table 3. Most individuals remained in the same category. Among those with higher levels of concurrent use (4 or more medicines), 381 individuals would have no concurrent medicines and 18,057 individuals would reduce to 2–3 medicines with overlapping ADR risk (Table 3).

**Table 3:** Change in number of medicines with falls/fracture ADR profile, including and excluding potentially inappropriate medicine (PIM)

Change in number of medicines (with → without PIM)	Number of people	Change in concurrent medicines with similar ADR profiles
0–1 → 0–1	96,726	No change
2–3 → 2–3	135,089	
≥ 4 → ≥ 4	59,924	
2–3 → 0–1	13,422	Reduced to no concurrent medicines
≥ 4 → 0–1	381	
≥ 4 → 2–3	18,057	Reduced to fewer concurrent medicines

Similarly, the number of older people with no overlapping ADR risk would increase from 89,615 to 109,011; an increase of 19,396 (Table 4). Most individuals remained in the same ADR risk type category.

**Table 4:** Change of total number of ADR types from concurrent medicines with overlapping ADR, including and excluding potentially inappropriate medicine (PIM)

Change in number of overlapping ADR types (with → without PIM)	Number of people	Change in concurrent medicines with similar ADR profiles
0 → 0	89,615	No change
1 → 1	19,832	
2–3 → 2–3	124,273	
≥ 4 → ≥ 4	38,418	
1 → 0	7,337	Reduction to no overlapping ADR
2–3 → 0	8,070	
≥ 4 → 0	3,989	
2–3 → 1	5,051	Reduction to fewer overlapping ADR types
≥ 4 → 1	3,839	
≥ 4 → 2–3	23,175	

## Discussion

Our findings demonstrate that concurrent use of medicines with overlapping ADR profiles is common among older adults, with potential medicine-related harm highlighted by the use of PIMs. Using data from the Australian PBS 10% sample, we found that 244,331 older adults were dispensed two or more medicines associated with at least one ADR type, which extrapolates to an estimated 2.4 million older Australians. With the national population of 4.2 million Australians aged 65 years and older [17], this suggests that approximately 57% of older Australians were dispensed concurrent medicines with overlapping ADR profiles. These findings are applicable to other healthcare settings with ageing populations, where polypharmacy and potentially inappropriate medicine use are widely reported [9, 10].

The most common overlapping ADR types identified in this study were falls and fractures, constipation, and renal injury. These ADR types are common and clinically important among older adults internationally. Falls are the second leading cause of unintentional injury deaths worldwide, with fatal falls occurring most frequently among adults aged 60 years and older [18]. Constipation is associated with significant healthcare utilisation, including substantial direct healthcare costs [19]. Acute kidney injury (AKI) also disproportionately affects older adults, particularly those aged 75 years and older, and is associated with increased short-term mortality [20]. Therefore, our findings indicate that overlapping ADR profiles arising from concurrent medicine use align with clinically important adverse conditions commonly observed in older populations, underscoring the importance of identifying medicines that contribute to cumulative risk in this population.

Identifying PIMs is a recommended first step in prioritising deprescribing opportunities [7]. This analysis demonstrated the potential population-level benefits of using explicit PIM criteria [15] to prioritise deprescribing, with a 6%, 13%, and 11% relative reduction in number of older adults exposed to concurrent medicines associated with risk of falls or fracture, constipation, and renal injury, respectively. Larger relative reductions were observed for some less prevalent ADR types; however, these should be considered together with the number of people affected when identifying priorities for clinical review and potential deprescribing decisions.

Our analysis provides an example of a data-driven, proactive deprescribing approach that seeks to identify medicines associated with potential harm before adverse patient outcomes occur. Increasing pharmacists’ data analytic capability has been highlighted as an essential yet underdeveloped area in current pharmacy practice, and our analysis demonstrates how routinely collected medicines data can be used to support pharmacists’ roles in medication safety and quality improvement [21].

A key strength of this study is the use of a large, nationally representative dispensing dataset to examine concurrent medicine exposure and overlapping ADR profiles at the population level. However, dispensing data reflect medicines supplied rather than medicines actually consumed; therefore, concurrent medicine exposure represents potential rather than confirmed exposure. In addition, dispensing data do not capture clinical indication or individualised treatment decisions, and some medicine combinations or PIMs identified may have been clinically appropriate for individual patients. Accordingly, PIM criteria were applied in this study to illustrate potential deprescribing opportunities, rather than to assess prescribing appropriateness. The analysis was based on a hypothetical scenario excluding PIMs and does not account for clinical feasibility, patient preferences, or potential unintended consequences of deprescribing. In addition, over the counter and hospital-supplied medicines were not captured, which may result in underestimation of total medicine exposure. Finally, ADR profiles were defined based on product information and pharmacological mechanisms and do not represent observed adverse events.

In this analysis, we used an adapted version of the 2018 Scottish cumulative toxicity tool [13] and the 2024 Australian PIMs list [15] as examples of a structured approach to assess the potential for medicine-related harm in older adults. While the tools provide a framework for identifying ADR risks from concurrent medicine use, other internationally recognised tools and methodologies, including the Beers Criteria and STOPP/START, are also widely used and are summarised in an existing handbook for multimorbidity and polypharmacy management [22]. Incorporating such tools into routine practice is likely to support safer medicines use in older adults, particularly those with multimorbidity and polypharmacy.

## Conclusion

Concurrent use of medicines with overlapping ADR profiles is common among older adults. Structured assessment tools may help identify potential deprescribing opportunities and areas of potential medicine-related harm. Further work is needed to evaluate how these approaches can be integrated into routine practice and their impact on patient outcomes.

## Data Availability

This study used the Pharmaceutical Benefits Scheme (PBS) 10% sample data, which are available for approved projects subject to approval by the Services Australia External Request Evaluation Committee (EREC).
